# ABUSE, NEGLECT, AND EXPLOITATION IN ASSISTED LIVING: AN EXAMINATION OF LONG-TERM CARE OMBUDSMAN COMPLAINT DATA

**DOI:** 10.1093/geroni/igz038.1777

**Published:** 2019-11-08

**Authors:** Karen Magruder, Ling Xu, Noelle L Fields

**Affiliations:** 1 Senior Source at Dallas, Senior Source in Dallas, Texas, United States; 2 University of Texas at Arlington, Arlington, Texas, United States

## Abstract

Adult Protective Services (APS) has seen cases of elder abuse increase recently and older adults living in long-term care facilities are subject to abuse, neglect and exploitation (ANE) at higher rates than community-dwelling seniors. However, there is scant empirical literature about ANE in assisted living (AL) settings and skilled nursing facilities (SNFs). The purpose of this study is to examine ANE complaints in AL and SNFs as recorded by Long-term Care Ombudsman Programs (LTCOPs) utilizing secondary data from a statewide database as well as an agency database used by staff ombudsmen who work in a large Metropolitan city in Texas. The sample included 140,497 complaints made at 1,940 licensed ALs with approximately 45,107 residents and 1,231 SNFs with 93,932 residents in Texas from 2010-2017. The percent of total complaints coded as ANE was higher in AL (2.01%) than in SNFs (1.46%) (p < .001). However, after controlling for number of residents, the rate of total ANE complaints per resident was higher (0.019) in SNF compared to AL (0.007) (z = -17.221, p <. 001). The incidence of financial exploitation was significantly higher in AL (23.46%) than in SNF (11.90%) (z = 5.582, p < .001). The percentage of verbal/psychological abuse in SNF (34.78%) was significantly higher than that in AL (23.4%) (z = -2.238, p < .05). Study findings contribute to the knowledge about the prevalence and nature of ANE in long-term care communities and bolster support for increased AL ombudsman presence, staff training, resident-centered care models, and AL oversight.

